# Targeting Aberrant Sialylation to Treat Cancer

**DOI:** 10.3390/medicines6040102

**Published:** 2019-10-13

**Authors:** Jennifer Munkley, Emma Scott

**Affiliations:** Institute of Genetic Medicine, Newcastle University, Newcastle upon Tyne NE1 3BZ, UK; Emma.Scott@newcastle.ac.uk

**Keywords:** glycosylation, glycans, cancer, sialic acid, sialylation, immunotherapy, therapeutics

## Abstract

Cell surface carbohydrates (known as glycans) are often aberrantly expressed or found at atypical levels in cancer. Glycans can impact all steps in tumour progression, from malignant transformation to metastasis, and have roles in all the cancer hallmarks. An increased understanding of glycans in the metastatic cascade offers exciting new therapeutic opportunities. Glycan-based targeting strategies are currently being tested in clinical trials and are a rich and untapped frontier for development. As we learn more about cancer glycobiology, new targets will continue to emerge for drug design. One key change in tumour glycosylation is the upregulation of cancer-associated sialylated glycans. Abnormal sialylation is integral to tumour growth, metastasis and immune evasion; therefore, targeting sialic acid moieties in cancer could be of high therapeutic value. Here, we summarise the changes to sialic acid biology in cancer and discuss recent advances and technologies bringing sialic-acid targeting treatments to the forefront of cancer therapeutics.

## 1. Introduction

All cells are coated with carbohydrates, known as glycans, which form a layer on the cell surface known as the glycocalyx [1]. Glycans are present in many different forms (including glycoproteins, proteoglycans and glycolipids) and build the basis for a universal language (the glycome) that is used for communication between cells [2]. Aberrant glycosylation is a universal feature of cancer cells, and it is well established that even small changes to the glycome can severely affect tumour cell biology [3,4]. Common cancer-associated glycome changes include aberrant sialylation, fucosylation, truncated O-glycans, and changes to N- and O-glycan branching [5]. Aberrant glycosylation is linked to all of the cancer hallmarks, and glycans can impact all steps in tumour progression, from malignant transformation to metastasis [4,6]. This makes cancer-associated glycans attractive therapeutic targets, and new technologies to study glycans have spurred a renewed interest in this area [7].

**Figure 1:**
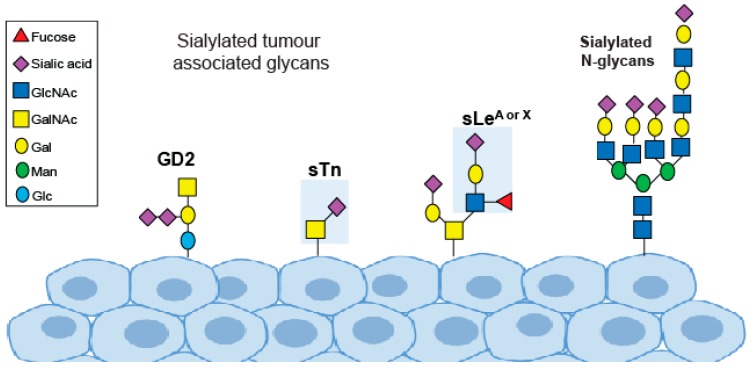
Sialylated glycans important in cancer.

One key change in tumour glycosylation is the upregulation of cancer-associated sialylated glycans, known as tumour sialoglycans [8]. Sialic acids are negatively charged sugar residues that often terminate the glycans of glycoproteins and glycolipids. The cell surface of cancer cells is covered with a dense layer of sialoglycans including SLeA, SLeX, STn and GM2 [9,10] (Figure 1). Aberrant sialylation plays a fundamental role in cancer cell growth, metastasis and immune evasion, and targeting sialic acid in cancer is an attractive therapeutic option. Here, we summarise the changes to sialic acid glycans in cancer and highlight recent advances in sialic acid therapeutics.

## 2. Tumour Growth and Metastasis

Aberrant sialylation contributes to tumour growth and metastasis at multiple levels and has been described as a key player in cancer progression [11,12,13]. Sialic acids can promote cancer progression by driving tumour growth, protecting cells from apoptosis, facilitating cancer cell detachment, enhancing invasion, promoting immune evasion, and enabling extravasation from the bloodstream to form metastases [8,10,14,15,16,17]. Sialylation of specific proteins has been linked to numerous signalling molecules and pathways important in cancer. For example, an increase in α2-3 sialylation in gastric carcinoma can lead to activation of the receptor tyrosine kinases MET and RON to promote a more invasive phenotype [18,19]. The terminal sialylation of N-glycans can also confer tumour cell resistance to hypoxia and have a major impact on malignant cell phenotypes [20,21]. N-glycans containing terminal α2-6 sialic acid contribute to the aberrant regulation of E-cadherin in cancer to impair cancer cell adhesion and promote cell invasion and metastasis [22,23,24].

New tools to target sialic acids are being developed and have shown promise in preventing/inhibiting cancer metastasis. The sialic acid glycomimetic (P-3Fax-Neu5Ac) delays tumour growth in vivo and can be delivered to cancer cells using nanoparticles to prevent the metastatic spread of melanoma cells to the lung in a murine model [25,26]. Intra-tumoral injections with Ac_5_3F_ax_Neu5Ac can block sialic acid expression and suppress tumour growth in multiple in vivo tumour models [10]. These findings demonstrate the important role of sialic acids in tumour growth and metastasis and indicate that a sialic acid blockade could be of high therapeutic value.

## 3. Glycosyltransferase and Glycosidase Enzymes

The tumour glyco-code is generated through the cooperative action of multiple glycosylation enzymes that catalyse the addition or removal of specific glycans onto proteins and lipids. These changes are often driven by the altered expression of genes involved in glycan synthesis [27]. Abnormal sialylation in cancer cells has been linked to the altered expression of both sialyltransferase and sialidase enzymes [28]. Sialyltransferases are enzymes that transfer sialic acid residues to the terminal position of glycoconjugates. The sialyltransferases are a family of 20 enzymes that catalyse the attachment of sialic acid to the underlying glycan chain via different glycosidic linkages (α2-3, α2-6, or α2-8). Sialyltransferases are expressed in a tissue-specific manner and can be further divided into four sub-families (ST3Gal, ST6Gal. ST6GalNAc, and ST8SIA) [29]. Sialyltransferases are often misregulated in cancer and this is linked to the expression of cancer-associated antigens that contain sialic acid [5,29,30].

Key examples of sialyltransferases important in cancer include ST6GAL1; ST3GAL4 ST3GAL6 and ST6GalNAc1/2 (summarised in Table 1). ST6GAL1 is upregulated in numerous cancer types and has recently been linked to all of the cancer hallmarks [11,31,32,33]. Therapeutically, ST6GAL1 can be used to predict response to EGFR/HER2 inhibitors in ovarian cancer cells [34]. Similarly, ST3GAL4 is also upregulated in cancer, and is associated with poor prognosis; metastasis and the synthesis of Sialyl Lewix X (sLe^X^) in gastric carcinoma [18,35]. ST3GAL6 is also linked to the synthesis of sLe^X^ and the generation of E-selectin ligands [36,37]. Recently, ST3GAL6 was found to be critical to bone marrow homing and resistance to therapy in multiple myeloma [36], and a follow-up study demonstrated this can be inhibited with the E-selectin antagonist GMI-1271 [37]. Other cancer-associated sialyltrasferases include ST6GalNAc1 (which catalyses the cancer-associated sTn antigen) and is associated with metastasis [8,27,38,39,40,41,42,43], and ST6GALNAC2, which has been identified as a metastasis suppressor in breast cancer patients and could be used to stratify patients for treatment with galectin-3 inhibitors [44,45].

The removal of sialic acids from glycoconjugates is catalysed by sialidase enzymes. Sialidases can modulate the binding sites of functional molecules and are important in various biological processes [46]. There are four sialidase enzymes, NEU1, NEU2, NEU3 and NEU4 and each of these can be altered in cancer [47,48,49,50,51]. Sialidase enzymes are emerging as novel therapeutic targets in cancer. Of particular interest, NEU1 can be targeted with oseltavimir phosphate in pancreatic, breast and ovarian tumours, and this may improve the drug sensitivity of chemoresistant cells [48].

## 4. Cancer-Associated Sialyloglycans

Changes to sialylated glycans in cancer include an upregulation of the Sialyl Lewis antigens (sLe^A^ and sLe^X^), an increase in the truncated O-glycan sialyl-Tn (STn) and an increase in the sialylated ganglioside GM2 [52] (Figure 1). The sialyl Lewis antigens are part of the Lewis family of blood group antigens, named after the discoverer of a series of antigens found on red blood cells, and are the minimal recognition motif for ligands of selectins (a family of lectins with roles in leukocyte trafficking and cancer metastasis). Tumour cells coated with sLe^A^ and sLe^x^ are recognised as migrating leucocytes, enabling them to escape the bloodstream and colonise other organs and tissues [53]. Many solid tumours and adenocarcinomas express high levels of sLe^A^ and sLe^X^, and thus targeting selectins and sLe^A/X^ is attractive therapeutically [53]. Potential strategies include the use of glycomimetic drugs, such as the selectin inhibitors Uproleselan (GMI-1271) and Rivipansel (GMI-1070), which have been tested in clinical trials [52,54] (Table 2). Uproleselan (GMI-1271) is in phase 3 clinical trials, in combination with chemotherapy, to treat relapsed acute myeloid leukemia (NCT03616470), and has also shown promise in pre-clinical models of breast cancer, where it can prevent bone metastasis and improve survival [55]. Rivipansel (GMI-1070) reached phase 3 clinical trials for sickle cell disease but reported negative results (NCT02187003).

One of the best characterised cancer-associated glycans is the truncated O-glycan STn, which is upregulated in virtually all epithelial cancers and linked to metastasis and poor patient outcome [8]. STn is carried by a variety of glycoproteins and glycolipids and has important role in tumour development and invasiveness [8]. The STn antigen has been investigated widely as a circulating biomarker for numerous cancer types [17], and a vaccine against STn has been tested in clinical trials and can increase survival in a subset of breast cancer patients receiving hormonal therapy [56,57,58].

Gangliosides (sialic-acid-containing glycosphingolipids) are important regulators of cell signalling in cancer [59]. The complex ganglioside GD2 is expressed on tumours of neuroectodermal origin and has a key role in the aggressiveness of some cancers including neuroblastoma and melanoma. GD2 can be inhibited with monoclonal antibodies and holds major potential as a target for cancer therapy [52,60]. The anti-GD2 monoclonal antibody dinutuximab can help improve survival in patients with high-risk neuroblastoma. Dinutuximab is currently in phase 3 clinical trials for neuroblastoma, and may be relevant to other cancer types [61].

## 5. Siglecs and Cancer Immunotherapy

**Figure 2:**
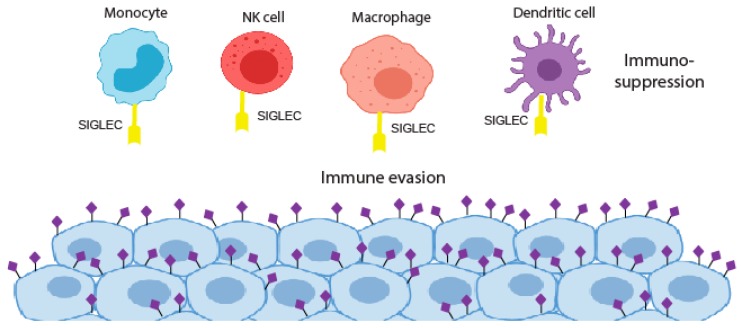
The siglec and sialoglycan axis in cancer. Siglec binding to hypersialylated tumour glycans blocks immune cell activation to promote immunosuppression.

The aberrant glycosylation of tumour cells can lead to new connections with immune cells resulting in an immunosuppressive phenotype. This can be through the induction of self-glycan structures to limit immune self-reactive responses or by the expression of glycans that can reduce the function of effector T cells [71]. The dense layer of sialic acids on the surface of cancer cells has long been implicated in protecting tumours from eradication by the immune system. In the 1960s and 1970s, immunotherapy clinical trials using irradiated cancer cells treated with bacterial sialidase were carried out but were not taken forward [72,73,74]. Recent developments in glyco-tools have driven a renewed interest in the role of sialic acids in the formation of an immuno-suppressive environment, with sialic acids on the surface of cancer cells believed to play a crucial role in immune modulation and tumour immune evasion [10]. Sialylated glycans found on both glycoproteins and glycolipids are recognised by Siglecs, a family of lectins that are expressed on the surface of many immune cell subtypes in the tumour microenvironment [75,76,77,78,79]. The interaction of cancer cell bound sialic acid with Siglecs can thus modulate immune cell phenotype and allow tumours to escape the immune system [64] (Figure 2). Cancer-associated glycans, such as STn and sialyl T, are well characterised examples of this, being linked to the impaired maturation and activation of macrophages and dendritic cells, and are also implicated in the deactivation of natural killer (NK) cells and the formation of regulatory T cells [63,78,80,81,82]. Hence, determining the specific glycan signature of cancer cells (known as the ‘glyco-code’) will be crucial to understand how glyans promote immune evasion [71,83].

Targeting sialic acid moieties has become an upcoming strategy for cancer immunotherapy [10,52]. Studies have shown that a sialic acid blockade has a profound effect on the tumour immune microenvironment, with pro-inflammatory effects, including increased numbers and activation state of CD8+ T cells, reduced percentages of myeloid and regulatory T (Tregs) cells, and increased tumour cell killing by cytotoxic t cells [10]. Bull et al. (2018) also demonstrated that a sialic acid blockade induces a protection upon rechallenge, suggestive of a curative immune response and conversion to a more immune-permissive microenvironment [10]. How sialic acid inhibition promotes these changes in tumour microenvironment is poorly understood; however, emerging studies suggest loss of tumour sialic acid can block the action of immune modulatory Siglecs on immune cells. To date, Siglecs-9, -7, -10 and -15 and their ligands have shown promise as targets to dampen anti-tumor immunity [62,63,64,65,66,67,68,69]. The interaction of Siglec-9 with sialylated MUC1 can induce the differentiation of monocytes to tumour-associated macrophages and increase levels of the checkpoint ligand PD-L1 [78,79]. Siglec-9 is upregulated on tumour-infiltrating T cells from patients with non-small lung cancer (NSCLC), colorectal and ovarian cancer and targeting of the sialoglycan-Siglec-9 pathway could be used to enhance T cell activation [63]. A high-profile 2019 study found that sialoglycoprotein CD24 acts an anti-phagocytic ‘don’t eat me’ signal that can protect cancer cells from attack by Siglec-10-expressing macrophages. Blockade of CD24-Siglec10 enhances clearance of CD24+ tumours and is a potrntial immunotherapy target [62]. Siglec-15 is upregulated in cancer cells and tumour-infiltrating myeloid cells, and is a critical immune suppressor. Siglec-15 suppresses antigen-specific T cell responses at the tumour site. An anti-Siglec-15 antibody has been developed and can reverse T cell suppression to promote tumour immunity and is potentially useful for cancer patients who are resistant to current therapies [69].

Precision glycocalyx editing with antibody-sialidase conjugates has also been reported and is a promising avenue for cancer immune therapy. Here, an antibody directs sialidase to selectively remove sialic acid from tumour cells and enable immune cells to kill the desialylated cancer cells [84]. The EAGLE platform (that delivers a targeted sialidase enzyme to the tumours) has shown great promise in pre-clinical models and is about to enter clinical trials for breast cancer [70]. Other potential strategies to target sialic acid glycans in cancer immunotherapies include anti-glycan vaccines, blocking cancer-associated glycan lectin interactions, and dendritic cell targeting [71].

## 6. Conclusions and Future Perspectives

In the era of personalised medicine, there is a huge potential to develop new glycan-based therapies to treat cancer. A key change in cancer glycosylation is an upregulation in the levels of sialylation, as well as the expression of cancer-associated sialoglycans. Aberrant sialylation is integral to tumour growth, survival, metastasis and immune evasion, and targeting abnormal sialylation will likely be of high therapeutic value. New technologies to study glycosylation are bringing glycan targeting strategies to the forefront of cancer therapeutics, particularly in the area of cancer immunology. Moving forward, drugs targeting tumour glycans will likely be used synergistically with existing chemotherapy and/or radiotherapy approaches to impact disease outcomes. As well as themselves being drug targets, changes to glycans can likely also be exploited to predict sensitivity and resistance to other treatment strategies and, ultimately, improve clinical outcome.

## Figures and Tables

**Table 1:** Key examples of sialyltransferases important in cancer.

Enzyme	Link to Cancer	Reference
ST6GAL1	Upregulated in numerous cancer types (including pancreatic, prostate, breast and ovarian cancer). Role in tumour growth and metastasis. Linked to several pathways intrinsic to tumour cell biology.	[11,31,32,33]
ST3GAL4	Upregulated in gastric carcinoma. Linked to poor prognosis, metastasis and the synthesis of sLe^X^.	[18,35]
ST3GAL6	High expression correlates with reduced survival in multiple myeloma. Influences homing and engraftment to the bone marrow niche in vivo. Plays a key role in selectin ligand synthesis through generation of sLe^X^.	[36,37]
ST6GALNAC1	Catalyses the sTn antigen and is associated with metastasis.	[8,27,38,39,40,41,42,43]
ST6GALNAC2	Metastasis suppressor in breast cancer. Could be used to stratify patients for treatment with galectin-3 inhibitors.	[44,45]

**Table 2:** Overview of pre-clinical models and clinical trials targeting aberrant sialylation in cancer.

Target	Approach	Reference or Identifier
Selectins	Selectin antagonist Uproleselan (GMI-1271) mimics SLeX. Uproleselan tested in pre-clinical models for breast cancer bone metastasis.	NCT03616470 phase 3 study in combination with chemotherapy for relapsed acute myeloid leukaemia. [55]
Sialylation	A sialic acid-blocking glycomimetic delivered using nanoparticles can inhibit metastasis and has been shown to be safe in pre-clinical models.	[26]
Siglecs	Siglecs-9, -7, -10 and -15 and their ligands have shown promise as targets to dampen anti-tumor immunity.	[62,63,64,65,66,67,68,69]
	The EAGLE platform (that delivers a targeted sialidase enzyme to the tumours) is about to enter clinical trials for breast cancer	[70]
sTn glycan	THERATOPE STn-KLH vaccine	NCT00003638 phase 3 clinical trial for metastatic breast cancer. [56,57,58]
Glycolipid GD2	anti-GD2 antibody ch14.18/CHO (dinutuximab)	Neuroblastoma phase 3 trial [61]
